# Spinal muscular atrophy type 1: A fatal case in a 1‐year‐old girl with delayed diagnosis

**DOI:** 10.1002/ccr3.8513

**Published:** 2024-02-09

**Authors:** Saira Batool Rizvi, Hafsa Ahmed, Arbaz Zaman, Ameenudeen Mohammed Nushrath Ali, Hussain Haider Shah, Sameer Abdul Rauf, Tirth Dave

**Affiliations:** ^1^ Dr Ruth KM Pfau, Civil Hospital Karachi Pakistan; ^2^ Dow University of Health Sciences Karachi Pakistan; ^3^ Department of Medicine Dow University of Health Sciences Karachi Pakistan; ^4^ Department of Internal Medicine Liaquat National Medical College Karachi Pakistan; ^5^ Bukovinian State Medical University Chernivtsi Ukraine

**Keywords:** delayed diagnosis, infant mortality, SMA Type 1, spinal muscular atrophy, Werdnig–Hoffmann disease

## Abstract

**Key Clinical Message:**

Spinal muscular atrophy (SMA) is a growing clinical concern, necessitating higher awareness and early detection. This case study focuses on the difficulties and advances in detecting and treating SMA. It emphasizes the value of early detection, interdisciplinary care, genetic testing, and novel therapeutics in terms of improving outcomes.

**Abstract:**

Spinal muscular atrophy type 1 (SMA Type 1) is a rare genetic neuromuscular disease characterized by muscle atrophy and weakness. This case report presents the fatal outcome of a 1‐year‐old girl with delayed diagnosis of SMA Type 1. The child exhibited symptoms of muscle weakness and respiratory distress, which were initially overlooked. Despite a thorough examination and diagnostic tests, including genetic analysis, SMA Type 1 with a homozygous deletion in the survival motor neuron 1 (SMN1) gene was confirmed. The child received supportive measures and physiotherapy but experienced a progressive deterioration of her condition and eventually succumbed to the disease. This case underscores the challenges of diagnosing SMA and highlights the importance of early identification for appropriate management. Improved awareness, diagnostic protocols, and access to treatment options, including pharmacological drugs and gene therapy, are essential to improve outcomes for SMA Type 1 patients, particularly in resource‐limited settings. Early detection through newborn screening programs and timely intervention can significantly impact the prognosis and life expectancy of SMA Type 1 children, emphasizing the need for continued research and clinical trials to establish a definitive cure.

## 1 INTRODUCTION

Spinal muscular atrophy (SMA) is a genetic neuromuscular disease characterized by muscle atrophy and weakness. The disease generally manifests early in life and is the leading genetic cause of death in infants and toddlers. SMA is caused by defects in the survival motor neuron 1 (SMN1) gene that encodes the SMN protein. The SMN protein is critical to the health and survival of the nerve cells in the spinal cord responsible for muscle contraction (motor neurons).^1^

Spinal muscular atrophy is an autosomal recessive genetic disease, meaning a person must have two copies of a defective gene to have the condition. SMA has generally been believed to affect as many as 10,000–25,000 children and adults in the United States; therefore, it is one of the most common rare diseases. One in 6000 to one in 10,000 children are born with the disease. The reported incidence is about one in 10,000 live births. The most severe form (Type 1) manifests before 6 months of age and generally results in death before age two.^1^, ^2^

Spinal muscular atrophy is a neurodegenerative disease characterized by progressive degeneration of alpha motor neurons, leading to muscle atrophy, paralysis, and even death. The clinical presentation of this disorder is hypotonia, symmetrical proximal weakness, atrophy, and reduced to absent deep tendon reflex. It is diagnosed by blood tests, genetic study, nerve conduction test (electromyography, EMG), and rarely muscle biopsy.^3^

Phenotypically, SMA has extreme variability and is clinically classified as SMA Type I (severe variant, Werdnig–Hoffmann) with the age of onset before 6 months, under which condition patient may not sit and usually die before their second birthday; SMA Type II (intermediate variant) with the age of onset between 7 and 18 months, patient may sit but never stand and death usually occurs after 2 years of age; in SMA Type III (mild variant, Kugelberg–Welander) with the age of onset being ~18 months of age, death occurs in the adult stage; and in SMA Type IV (adult variants) whose onset is in the second or third decade, individuals walk during the adulthood and death occurs in the adult stage. The clinical characteristics of the disorder are hypotonia, symmetrical proximal weakness, atrophy, and reduced to absent deep tendon reflexes. The gene for SMA, survival motor neuron (SMN1), has been mapped on the 5q11.2–13.3 region, and there are two copies of the SMN gene: SMN1 and SMN2.^4^

It is a rare but treatable disease, but its prevalence level is unknown in Pakistan due to a lack of awareness and diagnostic facilities. There are no data available about SMA patients in Pakistan. However, it is believed that the number is in the thousands due to the high rates of cousin marriages in Pakistan.^5^

We present a case of a 1‐year‐old girl experiencing coughing and suffering from respiratory distress for 2 weeks before admission. Upon further investigation, we discovered that the child's mother had noticed weakness in the child's lower limbs and had sought medical attention from multiple clinics, but the child was not fully diagnosed. At 8 months old, the child began experiencing respiratory issues and received only symptomatic treatment. After a thorough examination and various tests, the child was diagnosed with SMA Type 1.

## 2 CASE PRESENTATION

A 12‐month‐old girl with a BMI of 18.5 kg/m^2^ (weighing 6.9 kg and measuring 61 cm in length) was included in the study. She was born through cesarean section at 39 weeks of pregnancy with a birth weight of 2 kg and no history of decreased fetal movements or birth asphyxia. Although intellectually normal, the child is unable to achieve neck holding or sit, crawl, and stand due to muscle weakness. There is a family history of the same complaint of muscle weakness and respiratory distress in the paternal uncle, which led to the suspicion that the child's family history may be favorable for SMA. The mother observed hypotonia and muscle weakness in the child's legs, feet, and extremities at the third month, and she began to develop respiratory complaints at the eighth month, leading to multiple hospital admissions due to respiratory distress and pneumonia complaints.

**FIGURE 1:**
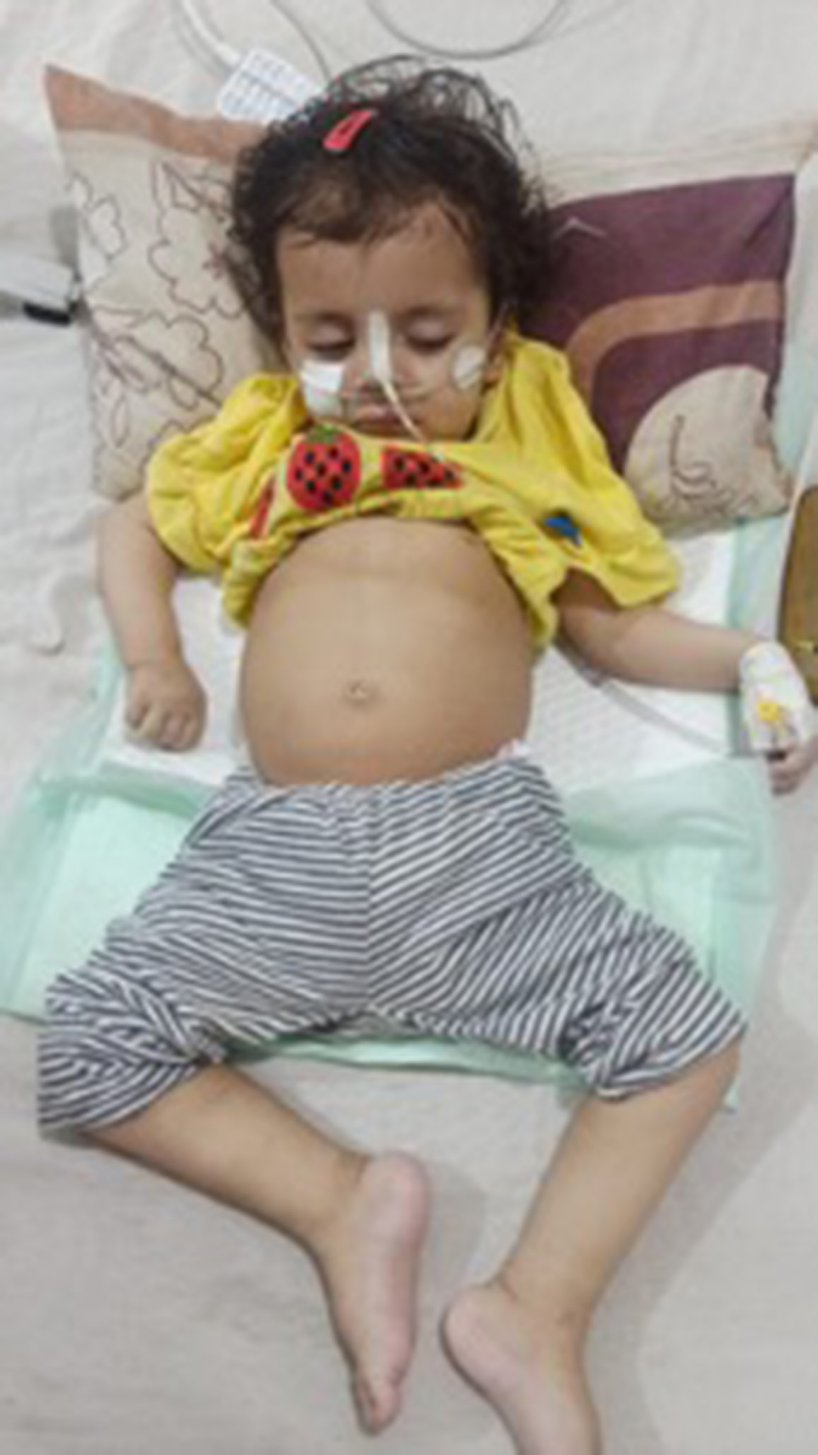
Patient with spinal muscular atrophy (SMA).

On May 9, 2023, the child was brought to the E.R. of Pediatrics‐II ward at Civil Hospital Karachi with cough and respiratory distress complaints for 2 weeks. She was initially admitted to the National Institute of Child Health for 2 days, after which she developed a non‐productive cough. She was given I.V. meropenem, I.V. vancomycin, and I.V. paracetamol for 7 days, and her chest CT showed pulmonary infection. On examination at Civil Hospital Karachi, the child was presented with signs and symptoms of respiratory distress, including harsh vesicular breathing, bilateral equal air entry, bilateral crepitus, and subcostal recessions. Her motor and sensory functions were assessed, revealing generalized hypotonia, weakness of limb muscles, absent reflexes, and tongue fasciculations. All baseline investigations were normal, but her chest radiograph showed air space shadowing with an air bronchogram involving the left middle and lower lobes. Figure 1 shows the patient with SMA.

## 3 METHODS

After multiple tests, including EMG, nerve conduction study, and creatine phosphokinase levels, the child was diagnosed with SMA Type 1, also known as Werdnig–Hoffmann disease, due to a homozygous deletion in the SMN1 gene with two copies of the SMN2 gene present. Although the child needed ventilator support due to severe respiratory distress, the parents did not give their consent. Therefore, the child was kept on the Do Not Resuscitate code and shifted to the high dependency unit, where she was kept on continuous positive airway pressure oxygen 6/6 cm H_2_O and given broad‐spectrum I.V. antibiotics to treat her pneumonia. The child was also given physiotherapy support, postural change, and frequent nebulization and suction to drain her mucus. Despite treatment for 20 days, the child did not survive.

## 4 DISCUSSION

This case report is unique and essential since SMA is a rare genetic disorder with a global prevalence of one in 6000–10,000 babies. Without effective treatment, most children die before the age of 2 years.^4^ Early treatment for SMA patients can considerably enhance motor function and quality of life. Phenotypes can be saved with early SMN restoration, ideally during the first three postnatal days. Early treatment initiation is critical for therapeutic outcome, yet the majority of SMA patients are diagnosed late, resulting in limited impact of available, life‐prolonging, and life‐saving therapies.^6^

Spinal muscular atrophy newborn screening programs in the United States, Taiwan, and Belgium can find afflicted children when they are asymptomatic, allowing for presymptomatic therapy before irreversible damage occurs. These treatments, however, are obtrusive and pose serious risks to the mother and unborn child. Noninvasive techniques, such as isolating fetal trophoblastic cells or cell‐free fetal DNA from maternal blood, have the ability to completely diagnose SMA. However, these procedures are costly and need specialized equipment, limiting their application in Pakistan.^6^

Some pharmacological drugs, treatments, and supportive care are available for SMA Type 1, which is the severe form of SMA. The cure for the disease is still not established, and treatment mainly consists of managing the symptoms and treating the complications. Moreover, in Pakistan, the prevalence of the disease is also not entirely known because most of the patients remain undiagnosed and unreported, as in this case report where the child was finally diagnosed at 1 year of age. As a result, the patients are not treated accordingly, leading to recurrent respiratory distress episodes and pneumonia, the most common cause of death among these patients.^7^

Several drugs have been approved by U.S. Food and Drug Administration (FDA) for treating SMA. These include nusinersin (Spinraza), which has been shown to increase the SMN protein that is needed for the maintenance of motor neurons and the drug risdiplam (Evrysdi), which can be used to treat SMA patients of age 2 months and older. A gene therapy technique, onasemnogene abreparivec‐xiii, delivers a fully functional human SMN gene to the targeted neuron via a virus to treat infantile‐onset SMA in children under 2 years of age. All these treatments and supportive care can be employed among SMA patients.^1^

Antisense oligonucleotides (Nusinersen, Spinraza) have been utilized to treat SMA patients after receiving FDA and EMA approval. In infantile‐onset SMA3, phase 3 trials revealed gains in motor function, event‐free survival, and HFMSE scores. Biogen released preliminary findings from a phase 2 trial of Nusinersen in presymptomatic SMA patients. Adenovirus‐mediated SMN1 gene replacement therapies, SMN2 splicing modulators, neuroprotective medicines, chemicals acting on muscles and neuromuscular junctions, and endocytosis, actin dynamics, and ubiquitin homeostasis modifiers are among the other intriguing approaches. Identifying affected children in the presymptomatic phase and projecting disease severity, on the contrary, are important challenges in introducing disease‐modifying medicines.^8^, ^9^

In Pakistan, diagnosis of and treatment for SMA children remain a challenge due to the lack of newborn screening programs for SMA and the high cost of US FDA‐approved SMA drugs. Our case report highlights the importance of these treatment options for patients and signifies the need for clinical trials to establish a definitive cure and the treatment for SMA children and find means of employing those treatment modalities in third world countries like Pakistan.^5^

Currently, SMA is only identified and treated when symptoms appear, and treatment frequently consists of supportive care and treating symptoms rather than the cause, which cannot be treated due to late diagnosis due to a lack of proper screening programs as well as the inability of diagnostic and treatment modalities to be affordable in Pakistan. Because SMA has a poor prognosis, especially Type 1, where baby usually dies before their second birthday, the condition is devastating for both the infant and their parents, who suffer emotionally as a result of SMA in their child.^10^ As a result, the regulations that promote the effective care of SMA children in Pakistan must be implemented.

According to the World Health Organization, if clinical genetic services in developing countries were significantly improved, 70% of birth defects could be prevented or cured globally. Prenatal or neonatal screening for genetic illnesses, for example, offers the opportunity to improve clinical care and prevent genetic conditions in the population. Despite the fact that a few nongovernmental organizations (NGOs) have recently initiated collaborative NBS (Newborn screening) efforts globally, Pakistan does not have a national public NBS initiative. The provision of genetic health care in Pakistan is complicated by a scarcity of medical genetics professionals. Furthermore, there are few rehabilitation and education programs in Pakistan for children with disabilities caused by genetic illnesses.^11^

Pakistan should consider developing a newborn screening program for genetic illnesses such as SMA in the future to guarantee early detection and treatment. Medical and genetic therapy for SMA children in government facilities require adequate financing. A proper healthcare infrastructure is also required, which includes genetic specialists, screening programs, testing, and treatment facilities. Collaboration with foreign funding agencies and NGOs to aid in the treatment and management of various ailments. This will aid in the early discovery and treatment of these uncommon genetic illnesses.^12^

Our case sheds light on the various management strategies, including supportive care, respiratory support, and physical therapy. This information can be valuable for clinicians who may encounter similar cases and need guidance on appropriate diagnostic approaches and treatment options. Therefore, considering the abovementioned case, it is desirable to first diagnose and report such cases in Pakistan and treat them accordingly to increase the survival ratio among SMA Type 1 patients.

## 5 CONCLUSION

In conclusion, this case report highlights the unique and essential nature of SMA, a rare genetic disorder with significant implications for affected children. The lack of effective treatment and the challenges in diagnosis and management make it crucial to raise awareness and establish appropriate protocols for identifying and treating SMA cases, particularly in countries like Pakistan where resources are limited. The availability of approved drugs and potential gene therapy offers hope for improved outcomes, but further research and clinical trials are necessary to establish a definitive cure. Early detection through newborn screening programs and prompt initiation of treatment can significantly impact the prognosis and life expectancy of SMA Type 1 children. This case underscores the importance of accurate diagnosis, comprehensive workup, and appropriate management strategies, providing valuable insights for clinicians faced with similar cases. Efforts should be made to diagnose, report, and treat SMA cases in Pakistan to improve the survival rate and quality of life for affected individuals.

## AUTHOR CONTRIBUTIONS

**Saira Batool Rizvi:** Conceptualization; data curation; writing – original draft; writing – review and editing. **Hafsa Ahmed:** Conceptualization; validation; writing – original draft; writing – review and editing. **Arbaz Zaman:** Writing – original draft; writing – review and editing. **Ameenudeen Mohammed Nushrath Ali:** Writing – original draft; writing – review and editing. **Hussain Haider Shah:** Supervision; writing – original draft; writing – review and editing. **Sameer Abdul Rauf:** Writing – original draft; writing – review and editing. **Tirth Dave:** Supervision; writing – original draft; writing – review and editing.

## FUNDING INFORMATION

None.

## CONFLICT OF INTEREST STATEMENT

None declared.

## ETHICS STATEMENT

The ethics approval was not required for the case report as per the country's guidelines.

## CONSENT

Written informed consent was obtained from the patient to publish this report in accordance with the journal's patient consent policy.

## Data Availability

The data that support the findings of this article are available from the corresponding author upon reasonable request.
